# Polar metabolomics using trichloroacetic acid extraction and porous graphitic carbon stationary phase

**DOI:** 10.1007/s11306-024-02146-7

**Published:** 2024-07-16

**Authors:** Francesca Day, Justin O’Sullivan, Farha Ramzan, Chris Pook

**Affiliations:** 1 Liggins Institute, The University of Auckland, Auckland, New Zealand; 2 The Maurice Wilkins Centre, The University of Auckland, Auckland, New Zealand; 3 MRC Lifecourse Epidemiology Unit, University of Southampton, Southampton, UK; 4 Australian Parkinson’s Mission, Garvan Institute of Medical Research, 384 Victoria Street, Sydney, Darlinghurst, NSW 2010 Australia; 5 A*STAR Singapore Institute for Clinical Sciences, Singapore, Singapore; 6 School of Chemical Sciences, University of Auckland, 23 Symonds St., Auckland, 1010 New Zealand

**Keywords:** Polar metabolites, Trichloroacetic acid, Porous graphitic carbon, LC–MS, HILIC, Untargeted metabolomics

## Abstract

**Introduction:**

Accurately identifying and quantifying polar metabolites using untargeted metabolomics has proven challenging in comparison to mid to non-polar metabolites. Hydrophilic interaction chromatography and gas chromatography–mass spectrometry are predominantly used to target polar metabolites.

**Objectives:**

This study aims to demonstrate a simple one-step extraction combined with liquid chromatography–mass spectrometry (LC–MS) that reliably retains polar metabolites.

**Methods:**

The method involves a MilliQ + 10% trichloroacetic acid extraction from 6 healthy individuals serum, combined with porous graphitic carbon liquid chromatography–mass spectrometry (LC–MS). The coefficient of variation (CV) assessed retention reliability of polar metabolites with logP as low as − 9. QreSS (Quantification, Retention, and System Suitability) internal standards determined the method's consistency and recovery efficiency.

**Results:**

The method demonstrated reliable retention (CV < 0.30) of polar metabolites within a logP range of − 9.1 to 5.6. QreSS internal standards confirmed consistent performance (CV < 0.16) and effective recovery (70–130%) of polar to mid-polar metabolites. Quality control dilution series demonstrated that ~ 80% of annotated metabolites could be accurately quantified (Pearson’s correlation coefficient > 0.80) within their concentration range. Repeatability was demonstrated through clustering of repeated extractions from a single sample.

**Conclusion:**

This LC–MS method is better suited to covering the polar segment of the metabolome than current methods, offering a reliable and efficient approach for accurate quantification of polar metabolites in untargeted metabolomics.

**Supplementary Information:**

The online version contains supplementary material available at 10.1007/s11306-024-02146-7.

## Introduction

Polar metabolites have important biological functions in energy metabolism, biosynthesis and signalling. Dysregulation of polar metabolite concentrations can manifest as disease phenotypes. For example, increased concentrations of branched-chain amino acids are positively correlated with obesity (Bagheri et al., 2019; Szczerbinski et al., 2022). Aspartate, glutamate, hypoxanthine, lactate, proline, and pyroglutamate concentrations are elevated in women with cervical cancer (Khan et al., 2019). Isoleucine, phenylalanine and tyrosine concentrations cumulatively correlate with increased risk of diabetes (Khan et al., 2019; Wang et al., 2011). The ability to easily and accurately quantify a large range of these compounds in readily accessible samples (e.g. blood) will enable identification of polar metabolites causatively or correlatively associated with diseases, providing evidence of potential mechanisms contributing to disease phenotypes.

Modern metabolomics seeks to identify and quantify all metabolites in a sample, whether that is relative to a structurally related and closely eluting internal standard, or absolutely to the abundance of a stable isotope labelled analogue (Roca et al., 2020). However, this is not currently possible due to the diversity of metabolite properties and abundance. The range of metabolite solubilities, the sensitivity and specificity of separation techniques available and ionisation abilities necessitate multiple methods to target non-polar (lipids), mid-polarity and polar metabolites, and those that only ionize in one or another polarity. Metabolite polarity mainly determines which metabolomic method is most suitable to accurately identify and quantify it (Roca et al., 2020). To capture the entire metabolome, it is necessary to use multiple methods, although this is difficult due to cost, and restrictions in time, instrument access and practical experience (Haggarty & Burgess, 2017).

Reverse phase liquid chromatography [RPLC] on in silica particulate columns with octadecylsilane stationary phase is the most reproducible and widely applicable chromatographic technique for metabolite identification and quantification (Zeki et al., 2020). Conventional RPLC retains and resolves metabolites with partition coefficient values higher than zero (log*P* > 0) i.e. mid- to non-polar metabolites (Dudzik & García, 2021; Roca et al., 2020). Chromatographic resolution of more polar metabolites (log*P* < 0) typically necessitates either hydrophilic interaction chromatography [HILIC] or derivatisation for analysis by gas-chromatography [GC] (Rakusanova et al., 2023; Roca et al., 2020; Zeki et al., 2020). In this paper we evaluate chromatographic resolution of polar metabolites using porous graphitic carbon [PGC] as a stationary phase. PGC exhibits increased retention of polar metabolites over C18 phases with RPLC, and shorter between-run stabilisation periods (Ramautar & De Jong, 2014). The extraction for PGC chromatography is simpler than the extraction methods required for HILIC (Ramautar & De Jong, 2014).

The high organic solvent starting conditions that are needed in HILIC require that samples are dissolved in a matched matrix. Whilst methanol and acetonitrile are commonly used to deproteinate metabolites for LC–MS-based analysis, it has long been known that this yields poor recovery of amino acids (Sedgwick et al., 1991). Aqueous solutions of perchloric or trichloroacetic acid [TCA] can be used as an alternative deproteinisation strategy to organic solvents. Such aqueous extracts are incompatible with HILIC, but they are compatible with RPLC and positive electrospray ionisation mass spectrometry. However, most RPLC stationary phases have limited or no retention of metabolites with log*P* < 0. Ortmayr et al. (2015) demonstrated that PGC columns can separate 30 polar metabolites that were not retained by RPLC, such as amino acids and sugar phosphates. Here we describe our work to explore the retention and separation power of this relatively underappreciated stationary phase for the resolution of the polar metabolome. We demonstrate the ability of a novel LC–MS method that is sensitive, repeatable and accurately identifies and quantifies a range of polar metabolites, offering a new solution for untargeted polar metabolomics.

### Objective

We set out to validate the performance of a novel LC–MS method with aqueous trichloroacetic acid extraction and porous graphitic carbon stationary phase.

## Materials and methods

### Ethics

Serum collection was approved by the Central Health and Disability Ethics Committee of the University of Auckland (20/CEN/69). The study was prospectively registered with the Australian New Zealand Clinical Trials Registry at www.anzctr.org.au (ACTRN12620000629932p).

### Solvents and chemicals

LC–MS grade solvents and modifiers were obtained (methanol, Merck; acetonitrile, Optima LC–MS grade; formic acid and ammonium formate, Thermo Fisher Scientific (Waltham, MA, USA)). Type 1 water was generated using a Millipore unit (Merck Millipore, Auckland, New Zealand). Trichloroacetic acid was obtained from Merck. QReSS (Quantification, Retention, and System Suitability) internal standard kit (cat no. MSK-*QReSS*-KIT) was obtained from Cambridge Isotope Labs Inc. (Tewksbury, MA, USA). Phosphate buffered saline [PBS] tablets and a standard mixture of 37 amines (Sigma product A9906) were obtained from Sigma Aldrich (now Merck KGaA, Darmstadt, Germany).

### Human serum samples

Serum was collected from six healthy individuals and stored at – 80 °C prior to analysis. Samples were de-identified prior to use. Three technical repeats were extracted from a single serum sample from each individual. An additional QC sample was created by combining equal parts of all six individual’s serum samples.

### Sample preparation

Pre-extraction, 10 µL of QReSS internal standard mixture (1.25 µL of QReSS internal standard1, 1.25 µL of QReSS internal standard2, 7.5 µL of water) was added to a 20 µL aliquot of serum in a 1.5 mL Eppendorf tube and vortexed (3s). To extract metabolites from the serum an internal standard mix, 50 µL of 90:10 mix of milliQ water [MQ] and TCA was added to the Eppendorf tube and vortexed (3s). The sample was incubated on ice (5 min), while protein precipitated. Samples were centrifuged (12,000 Relative Centrifugal Force [RCF], 5 min, 4°C). Following centrifugation, 50 µL of the supernatant was retained and diluted fourfold with MQ. Samples were vortexed (3s) prior to LC–MS analysis.

The Sigma A9906 amine standard was diluted ten times in PBS and extracted and analysed as the serum samples were.

The effects of the serum matrix upon linearity of the analysis was assessed via fractional dilution of serum extract with blank extract to 20%, 40%, 60% and 80% of the former.

### Liquid chromatography–mass spectrometry (LC–MS) conditions

LC–MS was performed using an Accela 1250 pump (Thermo Fisher Scientific, Waltham, MA, USA), a Thermo TriPlus autosampler maintained at 8°C (Thermo Fisher Scientific, Hampton, VA, USA), a HotDog5090 column oven and a porous graphitic Supel™ Carbon LC 2.7 μm HPLC Column (150 mm × 2.1 mm × 2.7 μm) maintained at 30 °C. Flow was 0.3 mL/min and the injection volume was 20 µL. The mobile phase (A) was MQ with 10 mM ammonium formate, 0.1% formic acid, mobile phase (B) was methanol with 10 mM ammonium formate, 0.1% formic acid, mobile phase (C) was acetonitrile with 0.1% formic acid.

The mass spectrometer was a Q-Exactive (Thermo Scientific, Dreieich, Germany) with a heated electrospray ionisation [ESI] source. Source voltage was 3.5 kV, S-lens RF level 50, heated capillary temperature 263 °C, AGC target 1^e6^ and resolution 17,500. Mass spectra were acquired in positive polarity and centroid mode. The scan range over the entire run was m/z 50–750 with stepped normalised collision energy (NCE) of 20, 30, 40 eV.

Data-dependent acquisition mode [DDA] was used to acquire data for qualitative analyses. The mobile phase gradient starts at 0% B and C for 1 min, then ramped to 0% B and 10% C over 2 min, then to 0% B and 30% C over 2 min, then to 0% B and 97% C over 3 min. The gradient returned to 10% B and 0% C for 1 min before being re-equilibrated to 0% B and 0% C from 9 to 15 min.

### Data processing

Chromatographically resolved features with MS/MS data were processed using MS-DIAL (v4.9) for peak picking, alignment, integration and annotation (Tsugawa et al., 2015). Features were annotated by reference to an in-house MS/MS library in .msp format based on the 2020 NIST HRMSMS library combined with the MS-DIAL VS15 library and standard spectra of the compounds in the QReSS kit and the Sigma A9906 amine mix, amongst various others (National Institute of Standards & Technology, Gaithersburg, MD, USA). Reference spectra from standards analysed in-house were manually exported from MS-DIAL and added to the .msp. Features in the data were annotated using the process internal to MS-DIAL which involves calculation of an aggregate score based on accurate mass similarity score of the putative adduct to the observed parent ion *m*/*z*, isotope ratios of the MS1 spectrum, spectral matching of MS/MS features using both dot product, reverse dot product, and the matched fragments ratio, and optional RT similarity score (not used here) (Tsugawa et al., 2015). The threshold for dot product was set at 0.75 as it has been shown that values lower than this significantly increase the false positive rate (Li et al., 2021).

Peak areas were blank-subtracted and normalised to recovery of the internal standard with the closest retention time. Ontological classifications for the annotations were scraped from the Human Metabolome Database v4.0 [HMDB] (Wishart et al., 2018), where present. Chromatographic performance metrics were calculated for all identified feature and internal standard peaks: Theoretical plate number by the Second Moment method (Cruz Villalon, 2023), tailing factor according to the International Pharmacopeia (WHO. 2008) and asymmetry factor according to the ASTM (ASTM International, 2020) (Supplementary Methods 1).

## Results

**Fig. 1:**
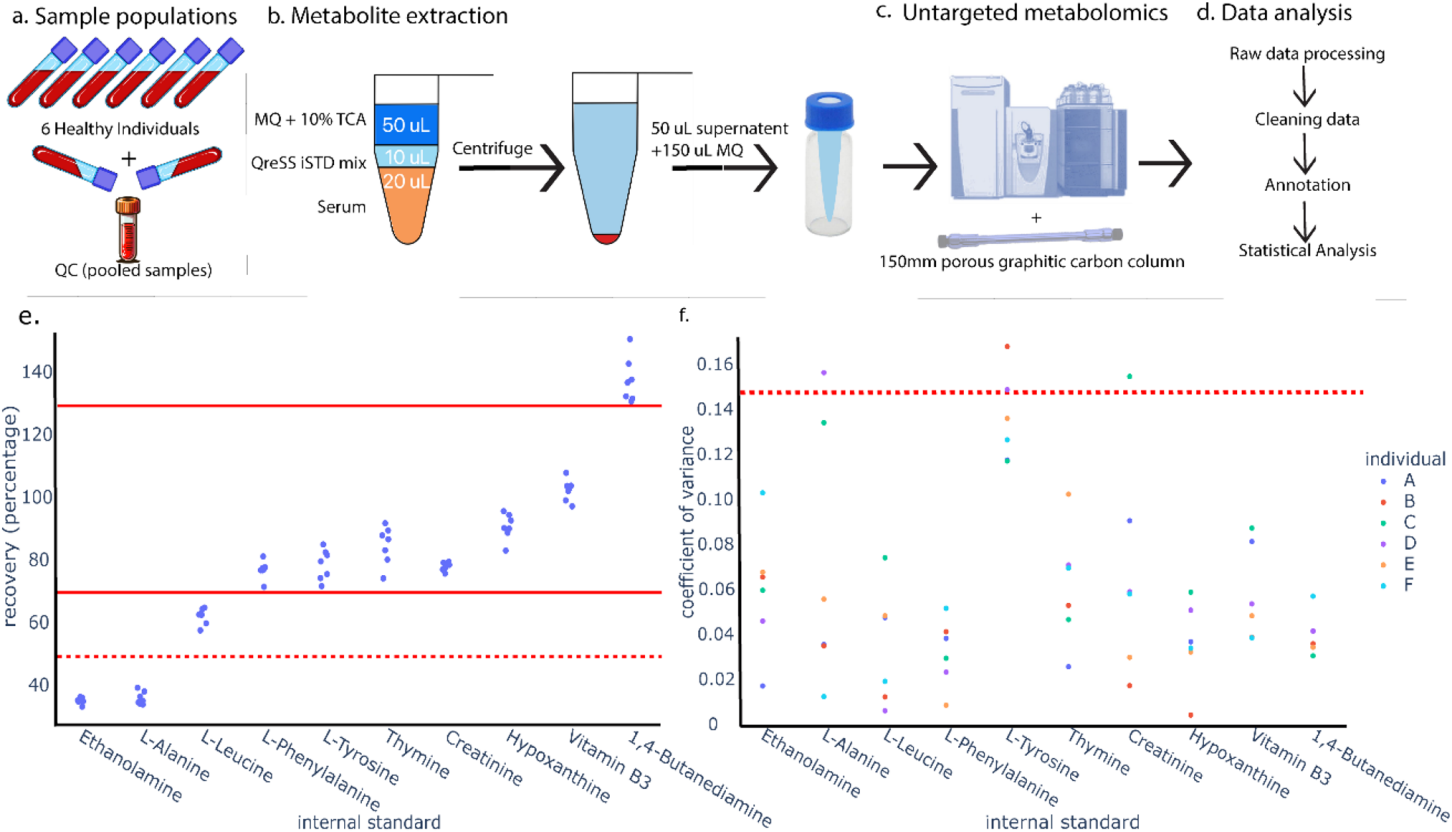
Polar metabolite methodology developed in this study. **a** Peripheral blood samples were obtained from six healthy individuals, and metabolites extracted from 20 µL of serum (methods). **b** Following centrifugation, the supernatant (50 µL) was transferred into an autosampler vial and diluted 1:3 with MQ H_2_O (150 µL). **c** Sample data were acquired by LC–MS/MS using a 150mm porous graphitic column and Q-Exactive MS using the data-dependent analysis mode. **d** Raw data was processed, cleaned, annotated and statistically analysed. **e** Recovery (%) of spiked internal QReSS standards was calculated using QReSS internal standard spiked blank (MQ) samples as a baseline for comparison. Samples that fall between the solid red lines represent reliably quantified metabolites. The dotted red line represents an alternative cut off (50% and above) for lowly but linear and repeatably recovered metabolites. **f** The CV of each QreSS internal standard, across 3 repeats from each individual. The dashed red line represents the CV threshold of 0.15 specified for targeted analysis by the United States Food and Drug Agency (FDA, 2018, 2019)

We observed 11 of the 16 compounds in the QReSS internal standard that ionise in positive mode (Supplementary Fig. 1). Tryptophan was not included in recovery calculations as its signal is lost rapidly in successive runs. Of the remaining 10 observed metabolites, six (i.e. creatinine, hypoxanthine, phenylalanine, thymine, tyrosine and vitamin B3) are recovered within the range of 70–130%, which is considered acceptable by targeted method validation criteria (Fig. 1e) (Alseekh et al., 2021; APVMA 2014). However, Alseekh et al. (2021) suggest that internal standards recovered up to 50%, if linear and reproducible, may be considered acceptable. QReSS alanine and ethanolamine recoveries were ~ 36%. Leucine was just below the threshold with 65% recovery. All detected internal standards had a mean Coefficient of Variation [CV] between 0.03 and 0.16. Labelled alanine and ethanolamine (< 70% recoveries) had mean CVs of 0.07 and 0.06, respectively.

**Fig. 2:**
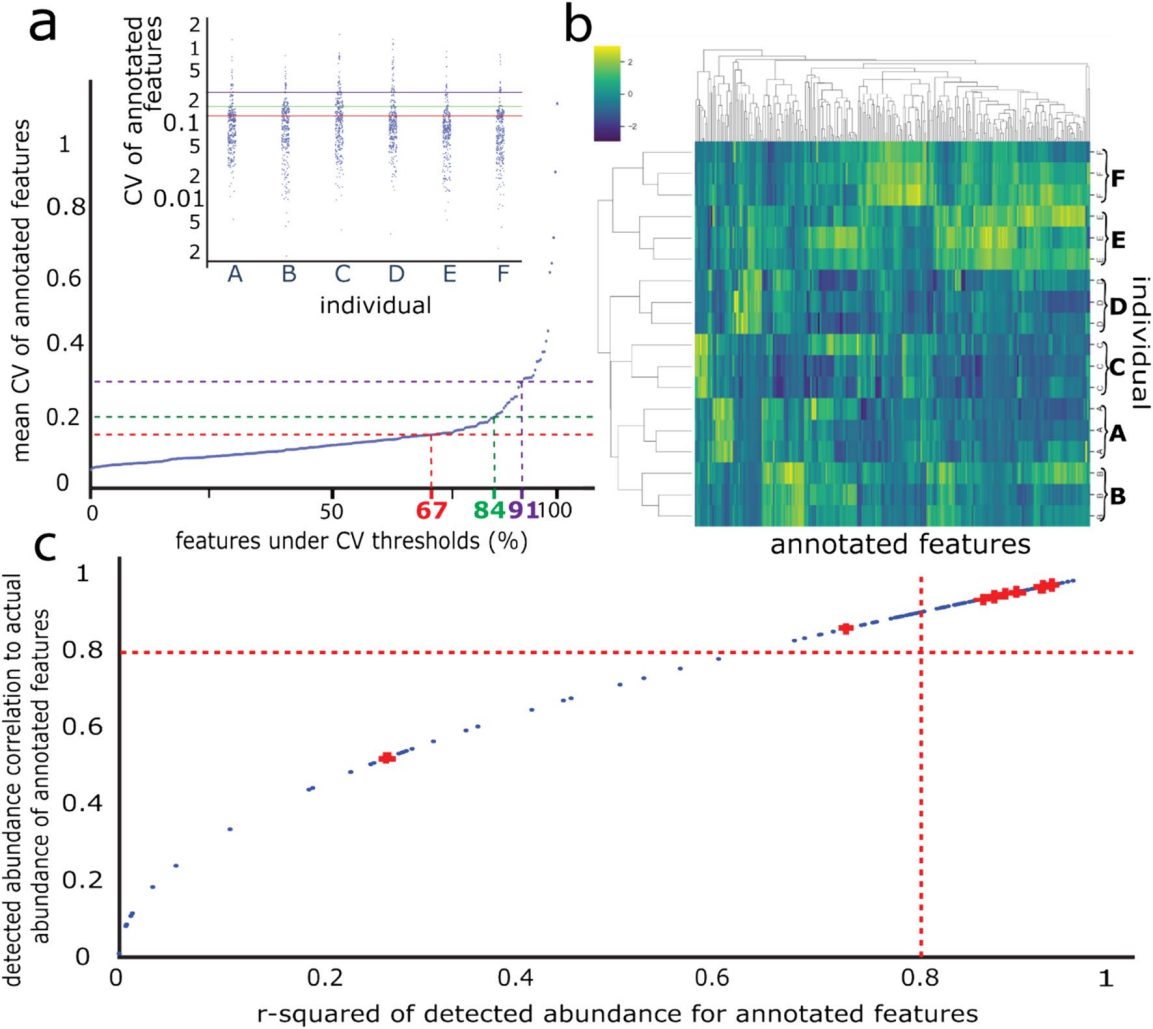
Visualisation of method performance. **a** The mean CV of each annotated metabolite, across 3 technical replicates from each individual, are plotted in ascending order. The smaller internal plot shows the CV of the 3 technical repeats of each annotated metabolite. Dashed lines represent the accepted thresholds for metabolomic methods: 0.15 (red, FDA approved), 0.20 (green, widely accepted) and 0.30 (purple, moderately accepted) (Brunius et al., 2016; Dudzik et al., 2018; FDA, 2018). **b** Cluster map demonstrating that the standardised abundances (colour intensity) of 274 annotated metabolites are more similar between technical replicates from each individual than between different individuals. **c** Relationship between the Pearsons correlation coefficient (correlation) and coefficient of determination (r-squared) value of detectable changes in metabolites, that were positively correlated, to actual concentration differences. Red crosses are the QReSS internal standard values. The red dotted lines show a threshold of 0.8

We detected 14,685 features and collected 1,907 MS/MS spectra that allowed us to annotate 274 features with a dot product (match) factor above 0.75. The percent of annotated features which met CV criteria 0.15, 0.20 and 0.30, were 66.8%, 83.7% and 90.9%, respectively (Fig. 2a).

We conducted a dilution series of post-extraction human serum supernatant to assess the linearity of the recovery response (i.e. coefficient of determination [R^2^] vs. Pearson correlation coefficient [PCC]) for each metabolite). The R^2^ value is a measure of the proportion of variance in the detected abundance of a metabolite that is explained by the actual change in concentration of that metabolite due to the dilution. The relationship between the R^2^ and PCC is quadratic, and both have specified threshold limits of > 0.80 for untargeted metabolomics (FDA, 2019). The PCC and R^2^ threshold was met by 79.2% and 68.0% of our annotated metabolites, respectively (Fig. 2c). Three metabolites had negative PCC values, in which absolute levels |R^2^| did not pass the > 0.80 limit (Supplementary Fig. 2). For example, theophylline had the largest negative PCC (− 0.64) from actual concentration increase to abundance detected. However, peak visualisation for theophylline identified multiple irregularly shaped peaks likely due to partial elution. The PCC for internal standards ethanolamine, 1,4-butanediamine, alanine, leucine, phenylalanine, vitamin B3 and thymine had high PCCs with values of 0.97, 0.94, 0.97, 0.95, 0.94 and 0.86. Tyrosine was lower, at 0.52. All internal standards had R^2^ thresholds above 0.87, except thymine (0.73) and tyrosine (0.27).

Clustering the samples according to metabolite abundance identified greater between individual variation than was observed for repeated extractions from the same serum sample (Fig. 2b).

**Fig. 3:**
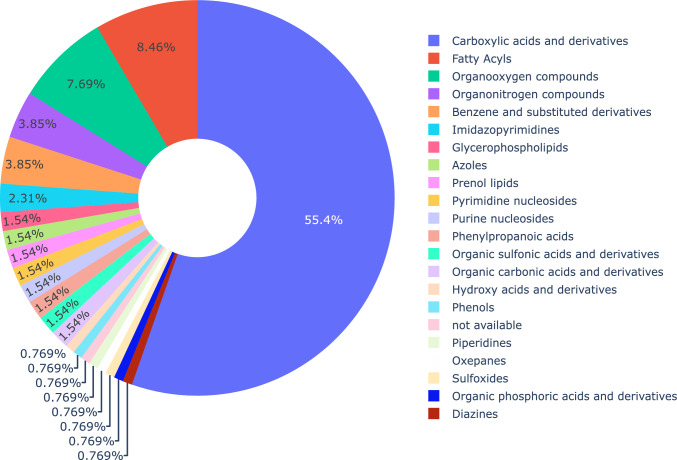
Pie chart summarising the relative proportions of annotated metabolite classes with entries in HMDB

Twenty-one metabolite classes were annotated using this method (Fig. 3). Most of these identified metabolites were carboxylic acids and their derivatives (55.4%). Sub-classifications within this class include amino acids, peptides, and analogues. By comparison, the next most abundant classes of compounds, fatty acyls occur at 8.46% of the annotated and class-identified metabolites, 7.48% are organooxygen compounds, 3.85% are organonitrogen compounds and 3.85% are benzene and substituted derivatives.

The mean (± SD) chromatographic performance values for the 52 features quantified here were 1.09e^6^ (± 2.01e^6^) for theoretical plate number, 1.22 (± 0.47) for tailing factor, and 1.36 (± 0.86) for peak asymmetry (Supplementary Table 2).

## Discussion

Polar metabolites represent an important fraction of the metabolome. Our novel RPLC–MS/MS method uses a PGC column to relatively quantify 14,645 features and absolutely quantify 40 features in human serum. MS/MS spectra were collected for 1907 features and 274 were identified to MSI level 2 and 40 to MSI level 1(Sumner et al., 2007). Fifty five percent of these annotations were carboxylic acids and derivatives. Triplicate analyses of six individual’s serum demonstrated that 90% of the annotated features had CVs lower than the widely used untargeted metabolomics threshold of 0.30 (Brunius et al., 2016).

Appropriate CV thresholds for metabolite analyses are determined according to the aim of the study. For example, less strict CV thresholds are typically applied to exploratory untargeted metabolomics analyses to increase metabolome coverage (Brunius et al., 2016). Thresholds for acceptable CVs for features in untargeted metabolomics vary but CVs between 0.20 (Dudzik et al., 2018) and 0.30 (Brunius et al., 2016) are generally accepted. Six internal standards (i.e. creatinine, hypoxanthine, phenylalanine, thymine, tyrosine and vitamin B3) had recoveries within 70–130% of the spiked blanks, five of these with CVs < 0.10. An additional four internal standards (i.e. leucine, alanine, ethanolamine and 1,4-butanediamine) met the acceptable CV < 0.15 for targeted metabolomics, specified by the United States Food and Drug Agency [FDA] (FDA, 2018). Thus, we claim the method we developed accurately and precisely quantifies polar metabolites in human serum relative to these internal standards.

Consensus criteria for the evaluation of the performance of untargeted metabolomics methods are still to be universally adopted. However, we recognise the comments of Dudzik et al. (2018), Kirwan et al. (2022) and Rakusanova et al. (2023) which include: the need to clearly communicate thought-out methodology; QCs and blanks to assess method consistency, spiked internal standards relevant to the targeted metabolites; and to keep the metabolite co-efficient of variation as low as possible. We incorporated these suggestions into our demonstration of the accuracy, reproducibility and utility of a novel LC–MS/MS method that identifies and quantifies polar metabolites. Our results compare well with results of HILIC or derivatisation-GC–MS methods (Rakusanova et al., 2023; Zeki et al., 2020). For example, Zhang et al. (2023) detected 755 positive ions in serum samples from 131 hypertensive children. The sample preparation required drying and reconstitution of the serum extract whereas our method involves a simple single extraction and centrifugation step, that can be completed within 15 minutes and immediately analysed. Furthermore, each sample takes only 15 minutes of LC–MS time to analyse, including re-equilibration of the column. Thus, our method is quick, easy and able to accurately and precisely identify a wide range of polar metabolites.

Aqueous TCA extraction and PGC LC resolve metabolites with log*P* values ranging from -9.1 (oxidised glutathione) to 5.6 (Supplementary Fig. 3). Approximately 70% of annotated metabolites captured by our method are polar, defined as log*P* < 0. This shows that PGC LC is complementary to bonded reverse phase stationary phases, which efficiently retain and resolve compounds with log*P* > 0. Due to acid–base reactions and mobile phase pH, amongst other factors, log*P* is not a direct correlate of retention time. A systematic investigation of structure-retention time relationships on PGC is beyond the scope of this study.

Chromatographic performance metrics are not well reported in untargeted metabolomics, despite peak shape and consistency being an important factor in obtaining accurate feature intensities and for ease in post-processing analyses (Brodsky et al., 2010). Where analysis of chromatographic performance had been done, the individual metrics are often incorporated into aggregate metrics and not reported in isolation (eg. Contrepois et al., 2015; Hosseinkhani et al., 2022). King et al. (2019) reported peak asymmetry values for nine features in their HILIC analysis. Our values compare well to theirs with kynurenine (vs their kynurenic acid), tryptophan, threonine, creatinine, and methyl-3-histidine exhibiting better asymmetry values. Our poorer asymmetry scores for alanine, leucine and hexose highlight a limitation of this method as several features exhibit peak splitting, particularly early-eluting, very polar metabolites, such as alanine, ethanolamine and serine. It should be stated that this limitation is a general one for untargeted analysis as the intention is to capture as much information as possible resulting in imperfect chromatography and matrix effects in the earliest part of the chromatogram and incomplete extraction and elution in the later parts. The void time, which is the time for an analyte that does not interact with the stationary phase to travel from the injector to the detector, was one minute for this analysis. Compounds that elute between 1 and 1.5 min, such as ethanolamine, were minimally retained and asymmetric (Supplementary Fig. 3). Putrescine and urea eluted at 1.6 and 1.7 min, respectively, and exhibited good symmetry. However, retention time alone did not determine good chromatographic performance. Other factors affecting peak shape and resolution include the prevalence of isomers. The hexoses, for example, elute together in a broad multiplet between 2.4 and 3.2 min. As the peaks were unresolved it is not possible to determine the relative abundance of glucose, galactose, fructose and mannose. Chromatographic performance plays an important role in post-processing analysis and obtaining accurate results in metabolomics analysis and so assessment and clear reporting of chromatography outcomes should be encouraged in future metabolomics studies to enable meaningful comparisons of performance (Brodsky et al., 2010).

In some cases, ion pair chromatography may also be used in polar metabolomics to improve peak shape, resolution and retention (Lioupi et al., 2023). A major limitation of this technique is contamination of the LC–MS with the ion pair reagents. This requires implementation of either robust cleaning and quality control procedures where instruments are to be used for non-ion-pairing analysis, or an instrument dedicated solely to such analysis. Additionally, ion pair chromatography is subject to adduct formation and ion suppression which can add further complexity to metabolomics (Lioupi et al., 2023).

Derivatisation-GC–MS is useful for polar metabolomics. However, derivatisation-GC–MS tends to detect fewer features than LC–MS and requires additional pre-processing steps that often impact sample integrity. For example, a recent GC–MS study detected 384 features (Eylem et al., 2022). Similarly, Rey-Stolle et al. detected only 241 features and of these 153 had a CV < 30 and 123 had a CV < 20 (Rey-Stolle et al., 2022). Furthermore, when used in conjunction with LC–MS to extend coverage to the non-polar fraction of the metabolome, GC–MS requires extra training and is costly (Haggarty & Burgess, 2017).

Previously, TCA as a metabolite extraction solvent in combination with NMR-based metabolomics was claimed to be a poor deproteinisation reagent that introduces artifacts (Daykin et al., 2002). We have demonstrated aqueous TCA to be an effective deproteinisation and extraction solvent for a wide range of polar metabolites and did not observe similar artifacts. Ninety percent of annotated metabolites, predominantly ‘carboxylic acids and derivatives’, were consistently recovered with a CV < 0.30. The disadvantage of using TCA is that it is a relatively strong acid (pKa 0.66) that can hydrolyse esterified compounds. Moreover, the high concentration of halogenated acid is inappropriate for ESI- modes, as it is anionic and quenches ionisation. Although ~ 80% of metabolites ionise to some degree in ESI + . Additionally, the water component of the TCA solution is more polar than methanol or acetonitrile and thus the process is less effective at extracting mid- to non-polar metabolites. However, this is advantageous when the mid- to non-polar metabolites are not the identified targets of the method. This is particularly true with PGC which irreversibly adsorbs molecules with planar saturated hydrocarbon moieties such as tryptophan.

Polar internal standards (i.e. creatinine, tyrosine, hypoxanthine, thymine and vitamin B3) were highly recovered by the paired TCA extraction and PGC chromatography method. By contrast, polar internal standards alanine and ethanolamine were not, possibly due to their loss during extraction, or ion suppression (Alseekh et al., 2021). Quantitation of creatinine, tyrosine, hypoxanthine, thymine and vitamin B3 were highly linear with abundance PCC values of 0.97 when compared to dilution, suggesting they are not entering the machine early enough to be affected by void enhancement or suppression. Tryptophan appears to be lost throughout the run possibly because it has an aromatic moiety that is strongly retained by the PGC, and only partially elutes. A stronger solvent mixture might resolve this, adding extra value to the method we have developed.

The QreSS (Quantification, Retention, and System Suitability) internal standard mix, consists of 18 metabolites labelled with stable isotopes to provide a mass delta of ≥ 3 Atomic Mass Units (Supplementary Table 1). The metabolites chosen span 10 metabolite classes representative of major central metabolism (Percy et al. 2024.). Their concentrations were designed to be within an order of magnitude of the physiological concentrations in plasma. The use of QReSS is a major strength of our study, particularly as the concentrations are at the lower end of their adult physiological quantities acquired from HMDB, demonstrating that the assay yields robust and biologically relevant information (Wishart et al., 2018). This is in part because metabolite identification and quantitation is restricted by the mass spectrometer dynamic range. Dynamic range is determined by metabolite abundance, methodology and instrument selection and can be tested by determining metabolite linearity by serial dilutions. Accurate quantification of metabolites is important to enable deciphering differences in metabolite concentrations between groups or individuals over time. Non-linearity can distort these differences to make them less or more significant than they are. Therefore, parametric tests, which use numerical value differences to determine significance, cannot be used on these metabolites. However, for those metabolites that are positively correlated (99.8%) non-parametric statistical tests that use ranks from observed data instead of the size of relative abundance difference, can be used to determine observable differences between samples.

Linearity is particularly important for internal standards, as these are used to normalise other metabolites and to reduce batch effects. Thus, non-linearity in internal standards may lead to distortions in the relative abundances of metabolites that are being normalised. QreSS internal standards, 1,4-butanediamine, alanine, leucine, phenylalanine, vitamin B3, and thymine had high observable r values to actual concentration therefore are reliable internal standards to use. However, tyrosine was relatively poorly correlated and is therefore less reliable as an internal standard for sample preparation and detectability. Interestingly tyrosine sensitivity seems to decline throughout the run, which would describe why its CVs are high in comparison to the other internal standards. This is likely due to tyrosine, like tryptophan, being aromatic.

Unsurprisingly as metabolite concentration is more highly correlated to detected abundance, the R^2^ of metabolite concentration is increased. The variance explained by the fitted regression model for 68.0% of annotated metabolites is higher than the acceptable 0.80 threshold, suggesting that the actual change in concentration for over two thirds of annotated metabolites is driving the differences we observed. Most internal standards sit above the 0.80 threshold and their detected abundance is relative to their actual concentration. Thus, the internal standards are reliable for normalisation across the tested concentration range.

‘Carboxylic acids and derivatives’, and ‘fatty acyls’ are the top classifications of our annotated metabolites that were identified in HMDB. These metabolites can be annotated to level 2 of the MSI guidelines (Sumner et al., 2007). Carboxylic acids are polar metabolites, while fatty acyls can be polar or non-polar dependent on the attached hydrocarbon chain. These classes have previously been identified in more complex LC–MS and derivatisation-GC–MS methods (Bian et al., 2020; Yu et al., 2021; Zhu et al., 2016). However, many metabolites were unable to be successfully annotated, and fewer than 50% (130/274) of them could be classified using the HMDB 2020 library. Increasing the number of annotated and classified metabolites would likely result in even greater diversity in our classifications.

Future developments of this method might incorporate stronger solvents to enable quantification of metabolites that strongly bind to the PGC. Further validation of this method using predefined metabolomic mixes (i.e. *credentialing* kit) may be used to show the MS/MS acquired features are not contaminants (Wang et al., 2019).

## Conclusions

Metabolite extraction using aqueous TCA, combined with PGC chromatography provides accurate and precise, qualitative and quantitative data on a range of biologically relevant polar metabolites in particular carboxylic and amino acids. We collated widely accepted thresholds and values to rigorously evaluate our method. Using consensus untargeted metabolomics criteria, more than 80% of the annotated features detected using this method can be quantified accurately and within their dynamic range. Importantly to our aim, most of these metabolites were polar. Further repeatability of this method was demonstrated through clustering of repeated extractions from a single sample. The use of spiked QReSS internal standards demonstrated the precision of recovery of most polar and some mid-polar internal standards. Those that were not well recovered may require a stronger mobile phase solvent to elute from the PGC. Even without these additional improvements, this LC–MS method is comparable, if not better suited to covering the polar segment of the metabolome than current methods. Researchers that want a simple and quick method to accurately and precisely, qualitatively and quantitatively detect the polar segment of the metabolome will find this method useful.

### Supplementary Information

Below is the link to the electronic supplementary material.

Supplementary file1 (DOCX 1903 KB)

Supplementary file2 (XLSX 31 KB)

## Data Availability

The raw data files generated in this study are available on request but cannot be shared outside the country of origin for reasons of data sovereignty.
