# Supplementary material for: Polar metabolomics using trichloroacetic acid extraction and porous graphitic carbon stationary phase
Source: Metabolomics. 2024 Jul 16;20(4):77. doi: 10.1007/s11306-024-02146-7 (PMC11252196; doi:10.1007/s11306-024-02146-7)
Supplement: Supplementary file 1 — Supplementary file1 (DOCX 1903 KB) [file 11306_2024_2146_MOESM1_ESM.docx]

**Supplementary methodology 1: Chromatographic performance metrics.**

**Theoretical plate number by the Second Moment method (Villalon, 2023).**

<https://pubs.acs.org/doi/full/10.1021/acs.jchemed.2c00588>

*N* = 2π × ( ( *t_r_* × *H* ) / *A* )^2^
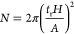


Where *t_r_* is retention time, *H* is peak height and *A* is peak area.

**Tailing factor according to the International Pharmacopeia (WHO, 2008)**

The ratio of the peak width at 5% height (*W*) to twice the front width (*f*), measured as the RT of the front edge at 5% height to the apex RT.

*T* = *W*_5%_ / 2*f*

**Asymmetry factor according to ASTM D8305−19**

<https://cdn.standards.iteh.ai/samples/105435/801755b7a5e54b3f805fdbc3b9f1929c/ASTM-D8305-19.pdf>

The ratio of the back peak width (*B*), measured from the RT of the apex to the RT of the back edge at 10%, to the front width (*A*), measured as the RT of the front edge at 10% height to the apex RT.

*A_S_* = *B* / *A*


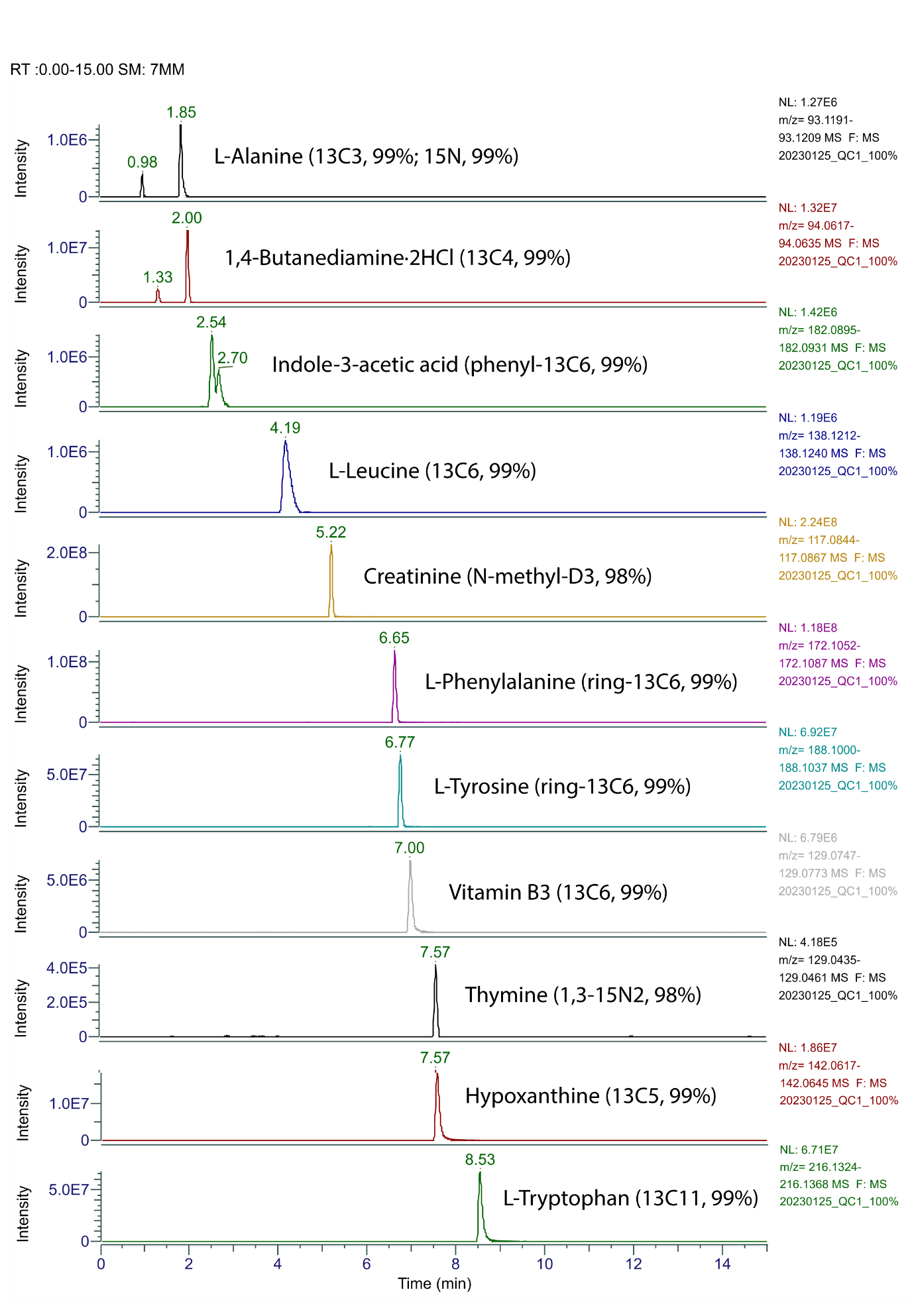

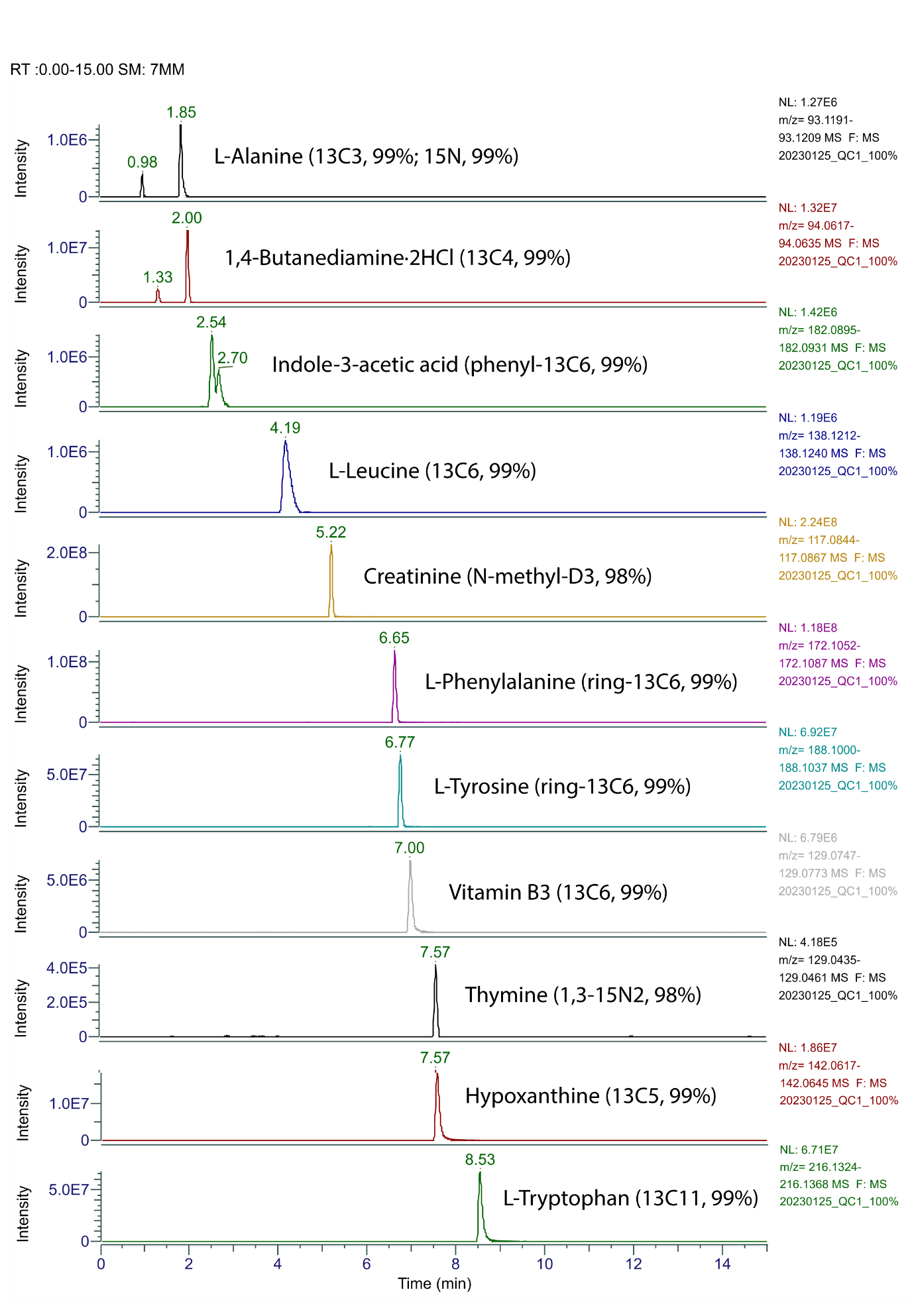


**Supplementary Figure 1.** LC-MS profile of spiked QReSS standards after extraction by extraction (A) and (B) solvent mix. Image produced in topview.

**Supplementary Figure 2.** Grid of selected EICs from feature peaks from each sample with a range of different shapes. Retention times in minutes is on the x-axis for each graph and the relative abundance of the features on the y-axis. a) kynurenine and b) alanine both have split peaks with a split of approximately 10:90 in relative abundance. c) tryptophan peak is gaussian although retention of tryptophan deteriorates quickly. d) hexose sugar peak is asymmetrical with two heads. e) threonine, f) creatinine and g) indole each have sharp gaussian peaks. h) leucine is gaussian although is affected by retention time shifting and i) methyl-3-histidine is asymmetrical with two heads, indicating the start of isomer chromatographic resolution.


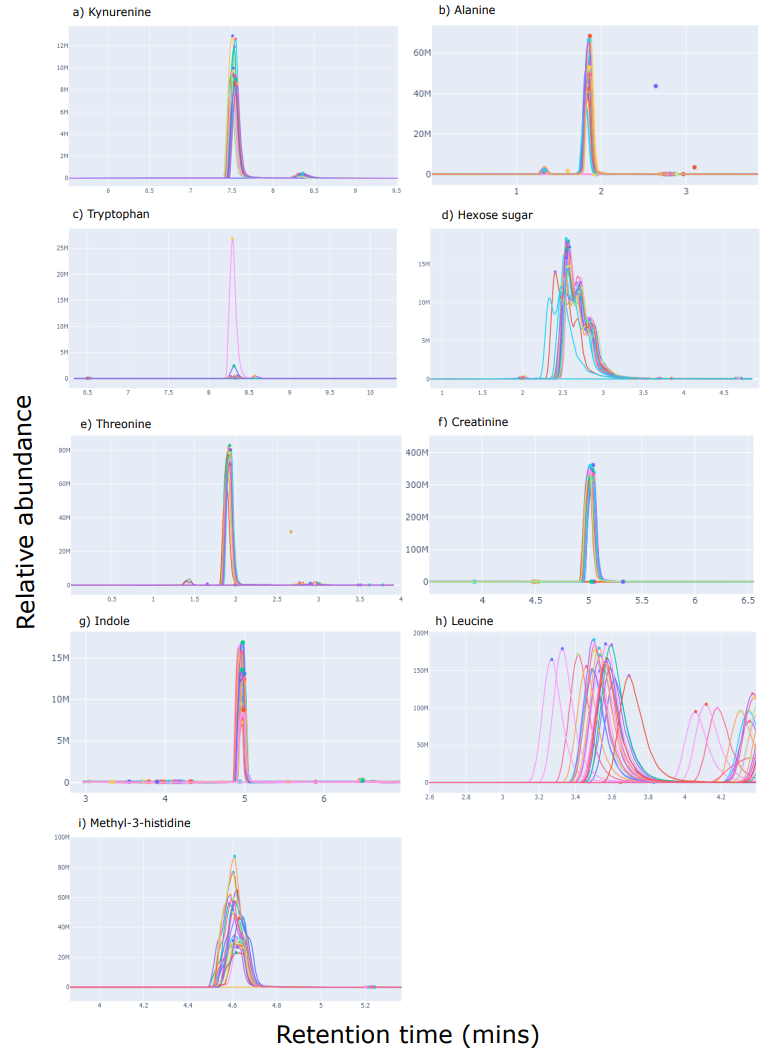


Leucine

Isoleucine

**
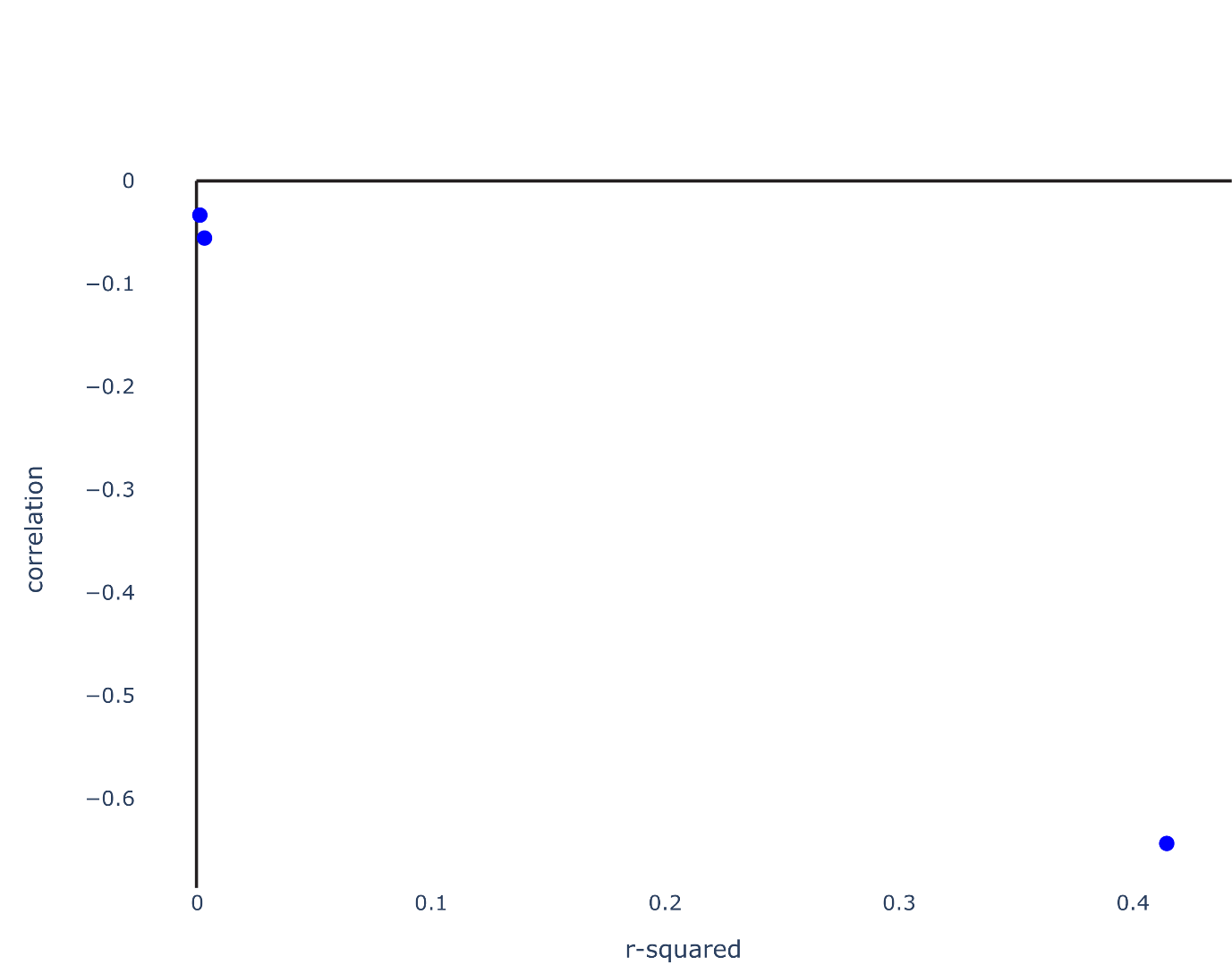
** **Supplementary Figure 3.** Relationship between the Pearson correlation coefficient (correlation) and coefficient of determination (r-squared) value of detectable changes in metabolites to actual concentration differences.

**Supplementary Figure 4.** The relationship between the partition coefficient (log*P*) and retention time of QreSS internal standards is positive shown clearly by the Pearson’s coefficient of correlation (R^2^), showing metabolites with lower log*P* values tend to be more weakly retained on the column.


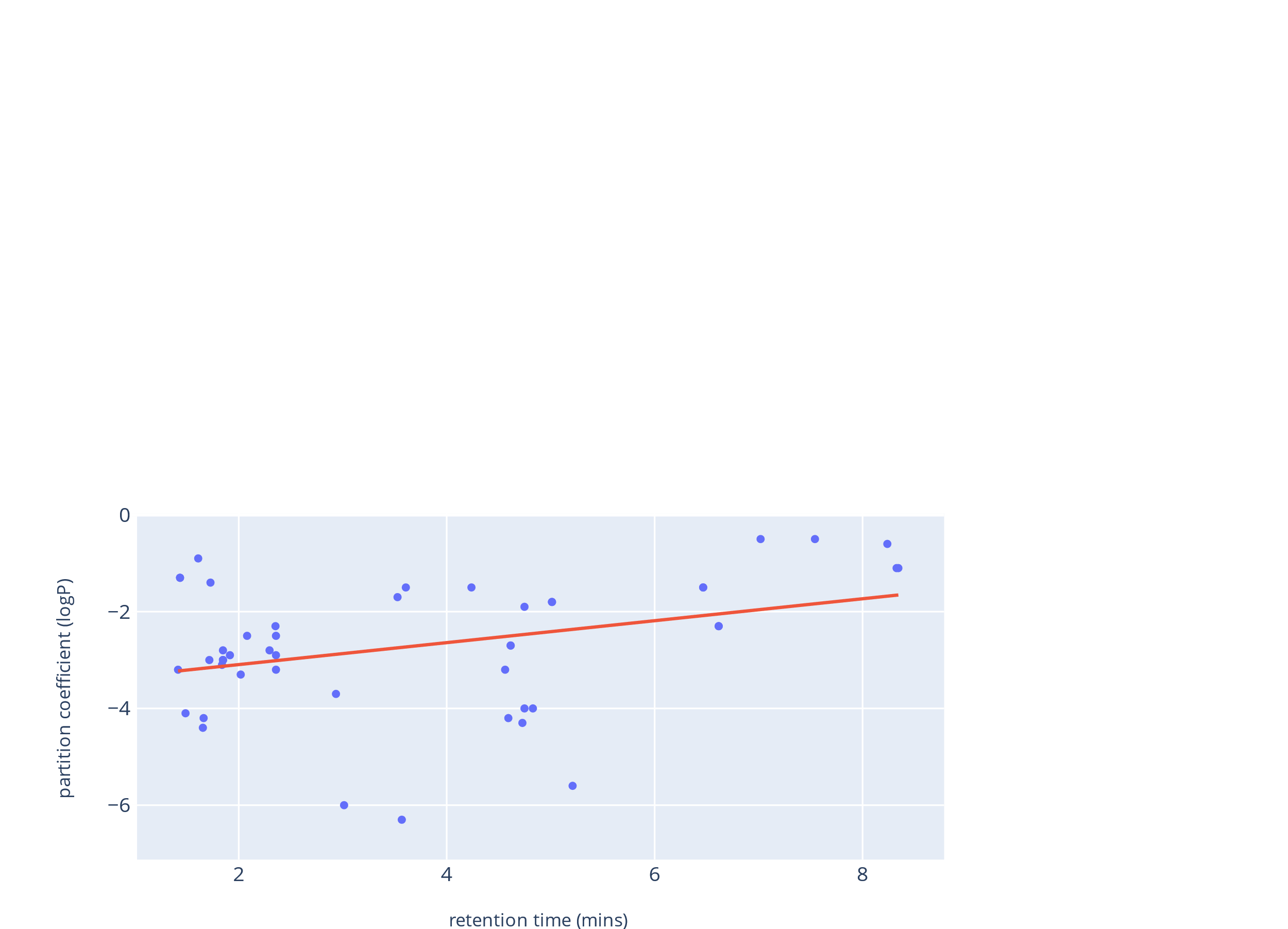


R^2^ = 0.12
